# Truncal changes in children with mild limb length inequality: a surface topography study

**DOI:** 10.1186/s13013-018-0173-z

**Published:** 2018-12-18

**Authors:** Theodoros B. Grivas, Konstantinos Angouris, Michail Chandrinos, Vasilios Kechagias

**Affiliations:** 1 Orthopaedics and Traumatology Department, “Tzaneio” General Hospital of Piraeus, Piraeus, Greece; 2 Orthopaedics and Traumatology Department, General Hospital of Ilia, Amaliada, Greece; 3 Orthopaedics and Traumatology Department, “Achilopoulion” General Hospital of Volos, Volos, Greece

**Keywords:** Limb length inequality (LLI), Leg length discrepancy (LLD), Scoliosis, Pelvic imbalance, Trunk imbalance, Trunk inclination, Spinal posture, Surface topography

## Abstract

**Background:**

Limb length Inequality (LLI) in children and adults may affect posture, gait, and several truncal parameters, and it can cause spinal scoliosis. In literature, however, there is a paucity of assessment of truncal and spinal changes due to mild LLI in children. This report presents children with LLI, and it aims to provide information in pelvic imbalance, spinal posture, and scoliotic curve, using surface topography analysis which is a novel methodological approach for this condition.

**Study design:**

This is an ongoing prospective research study on patient series suffering LLI.

**Material and method:**

Twenty children, attending the Scoliosis Clinic of the department, 7 boys, 13 girls, 9–15 years old, range 7.5–15, mean 15.5 years, having mild LLI, were assessed. The LLI was 0.5 to 2 cm, mean 1.2 cm. There was not any post-traumatic LLI. We evaluated the LLI in correlation to pelvic and spinal posture parameters. The 4D Formetric DIERS apparatus (4DF) was used for the surface topography assessment. The following were assessed: *in the coronal plane*, the coronal imbalance, the pelvic obliquity, the lateral deviation, and the 4DF scoliosis angle; *in the sagittal plane*, the sagittal imbalance, the 4DF kyphotic angle, the kyphotic apex, the 4DF lordotic angle, the lordotic apex, the pelvic tilt, and the trunk inclination; and *in the transverse plane*, the pelvis rotation, the pelvic torsion, the surface rotation, and the 4DF vertebral rotation. LLI was measured using a tape. The data were statistically analyzed, and reliability study for the LLI was also performed.

**Results/discussion:**

The LLI was statistically significantly correlated to the 4DF reading of pelvis rotation, pelvic tilt (pelvic obliquity), and surface rotation. The scoliometer readings (angle trunk rotation ATR or trunk inclination ATI) in the lumbar region were statistically significantly correlated to the 4DF readings of pelvic tilt (pelvic obliquity). The normally symmetric truncal parameters were also statistically significantly changed (all these deviating from the line of gravity through the vertebral prominence). Interestingly, LLI was not correlated to the scoliosis angle and the scoliometer reading at the lumbar level.

The following 4DF readings are presented: in the coronal plane, the coronal imbalance, pelvic obliquity, lateral deviation, and 4DF scoliosis angle; in the sagittal plane, the sagittal imbalance, kyphotic angle, kyphotic apex, lordotic angle, lordotic apex, pelvic tilt, and trunk inclination; and in the transverse plane, the pelvic rotation, pelvic torsion, surface rotation, and vertebral rotation.

**Conclusions:**

Previous studies have reported the results after simulation of LLI in order to evaluate the effects on the pelvic balance and spinal posture parameters. This report is not a LLI simulation study but it presents the effects of mild LLI on truncal changes in the main cardinal planes in children suffering LLI. These changes undoubtedly affect not only the standing truncal posture but also the gait’s economy as well.

As mild LLI affects the pelvic balance and spinal posture parameters, our therapeutic approach is that mild LLI (less than 2.0 cm) has to be corrected using shoe elevation, in order to equalize the pelvic obliquity and, consequently, the spinal posture parameters.

## Introduction

A *leg length inequality* (*LLI*) *or discrepancy* is a difference between the lengths of the legs.

LLI can be subdivided into two etiological groups: a structural LLI (SLLI) which is associated with a shortening of bony structures (congenital or acquired) [1] and a functional LLI (FLLI) [1, 2] which is the result of altered mechanics of the lower extremities, muscle, or joint contracture. In addition, persons with LLI can be classified into two categories: those who have had LLI since childhood and those who developed LLI later in life.

Several reports presented the effects of LLI on idiopathic scoliosis [3] and pelvic imbalance progression, low back pain, osteoarthritis of the hip, stress fractures, aseptic loosening of hip prostheses in adults, forces admitted through the hip, standing balance, and walking and running energy consumption [2, 4–8].

LLI critically affects the dynamics of lower limb growth, and it reflects the discrepancy in the growing skeleton. Asymptomatic LLI is relatively common in healthy pediatric and adult populations. Several studies of asymptomatic children or adults with LLI more than 2.0 cm report scoliosis and pelvic asymmetry [8, 9].

The correlation between LLI, scoliosis, and pelvic imbalance has been previously assessed in various ways, mostly on the basis of simulating LLI [10] and studying the consequences in trunk, spinal, and pelvic posture [4, 11–13]. These parameters regress with the equalization of LLI [7].

Management of LLI ranges from no treatment to multiple and specific procedures of limb length equalization [14].

In children, LLI is usually a parents’ observation or a physical finding identified incidentally during screening examinations as for idiopathic scoliosis [14].

For mild LLI (less than 2.0 cm), there is a lack of evidence in the literature. In this study, we present 20 children with mild limb length inequality aiming to investigate its impact to pelvic imbalance, spinal posture, and scoliotic curve, using Surface Topography Analysis. We consider this report a novel methodological approach study for this condition.

### Study design

This is an ongoing prospective research study on series of patients suffering LLI.

## Material and method

### The examined children

Twenty children, attending the Scoliosis Clinic of the department, 7 boys, 13 girls, 9–15 years old, range 7.5–15, mean 15.5 years, having mild LLI, were assessed. The LLI was 0.5 to 2 cm, mean 1.2 cm. There was not any traumatic LLI.

### Ethical issues

We obtained IRB approval (Ref#, the hospital IRB 50th meeting, 8-10-2015) and parental consent to participate and publish.

### The examining apparatus used

**Fig. 1:**
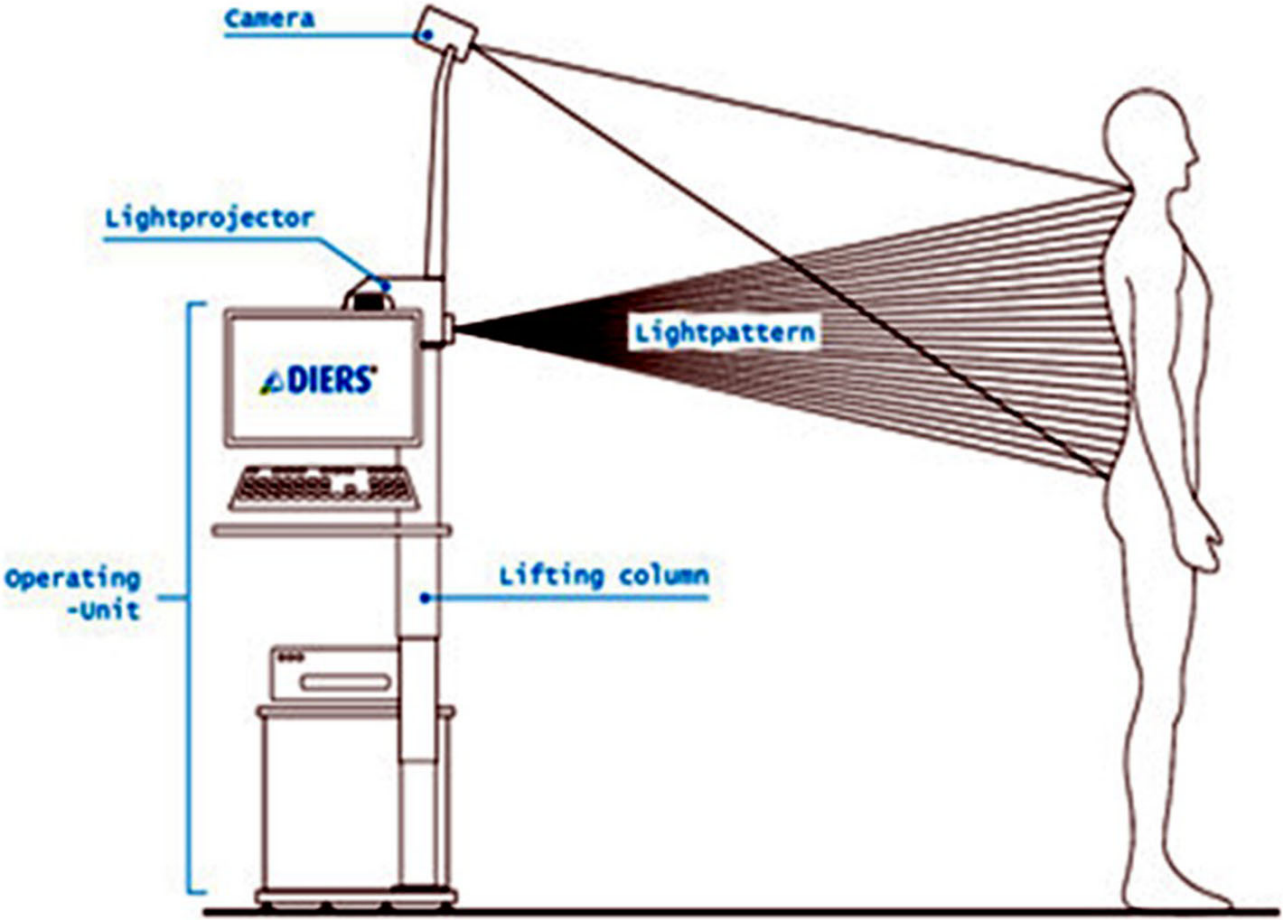
Rastersteography (surface topography) using the apparatus 4D Formetric DIERS (4DF)

The apparatus 4D Formetric DIERS (4DF) featuring a proper Software (FDA approved) was used for the measurements, and the reading resulted from the 4DF computation system. The Formetric apparatus functions by emitting a harmless white light (laser) to deliver a fast (40 ms), high-definition optical measurement of the surface of the back to produce graphical, clinical, and analytical information on the spine, the pelvis, and posture—without the need for radiation or invasive measures. This non-invasive, harmless imaging allowed us to reduce x-ray exposure of the examined patients Fig. 1.

### The 4DF measurements

**Fig. 2:**
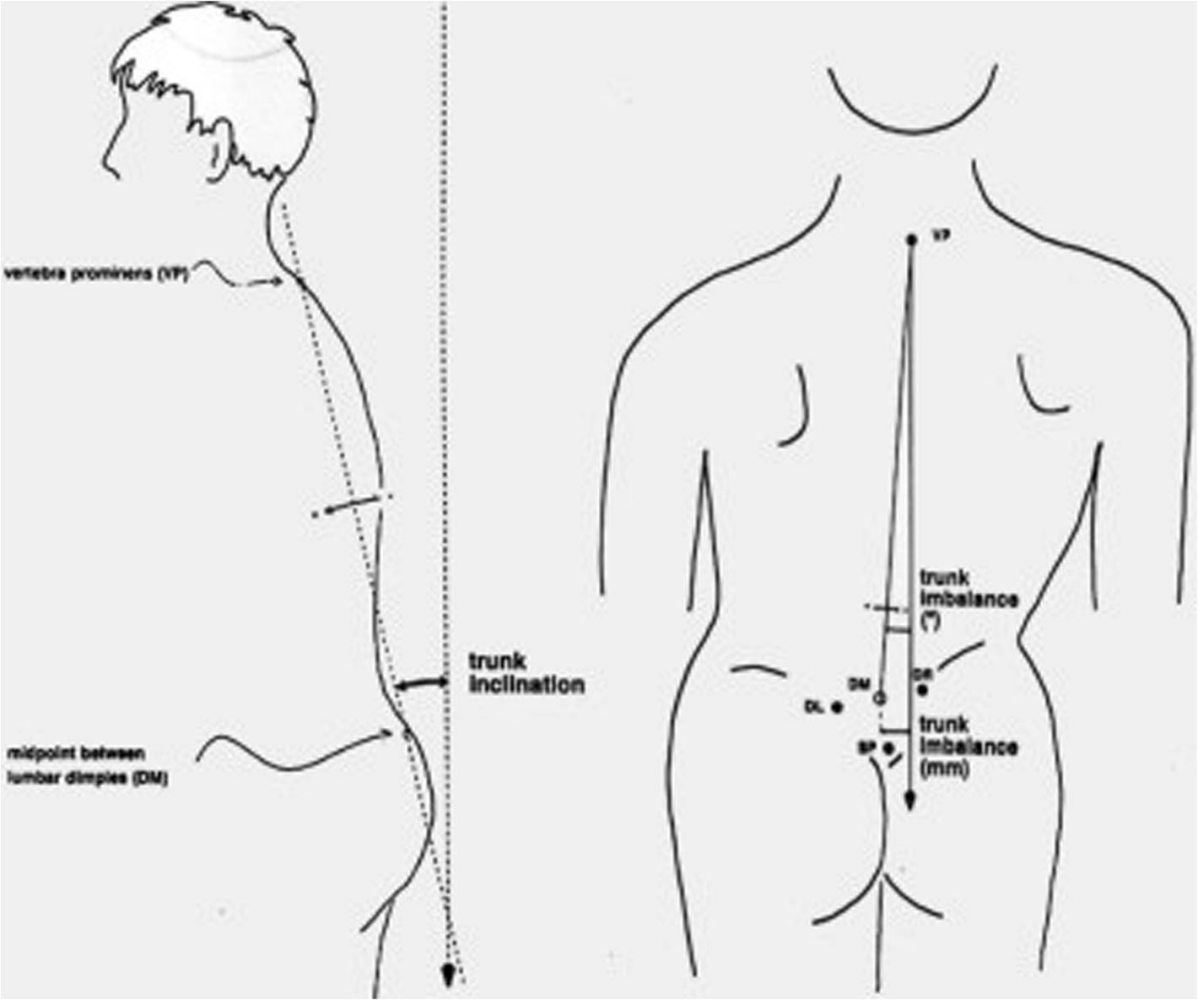
(From 4D Formetric Diers manual with permission). The dimple (D) distance is defined as the spatial distance between dimple left minus dimple right (DL-DR). The dimple middle DM lies on the center of the straight line connecting DL−DR. Trunk imbalance: the trunk imbalance is defined as the lateral deviation of vertebra prominence (VP) from DM *in millimeters*. Trunk inclination: the trunk inclination *refers to an angle* formed by the VP−DM line and the vertical in the sagittal plane

**Fig. 3:**
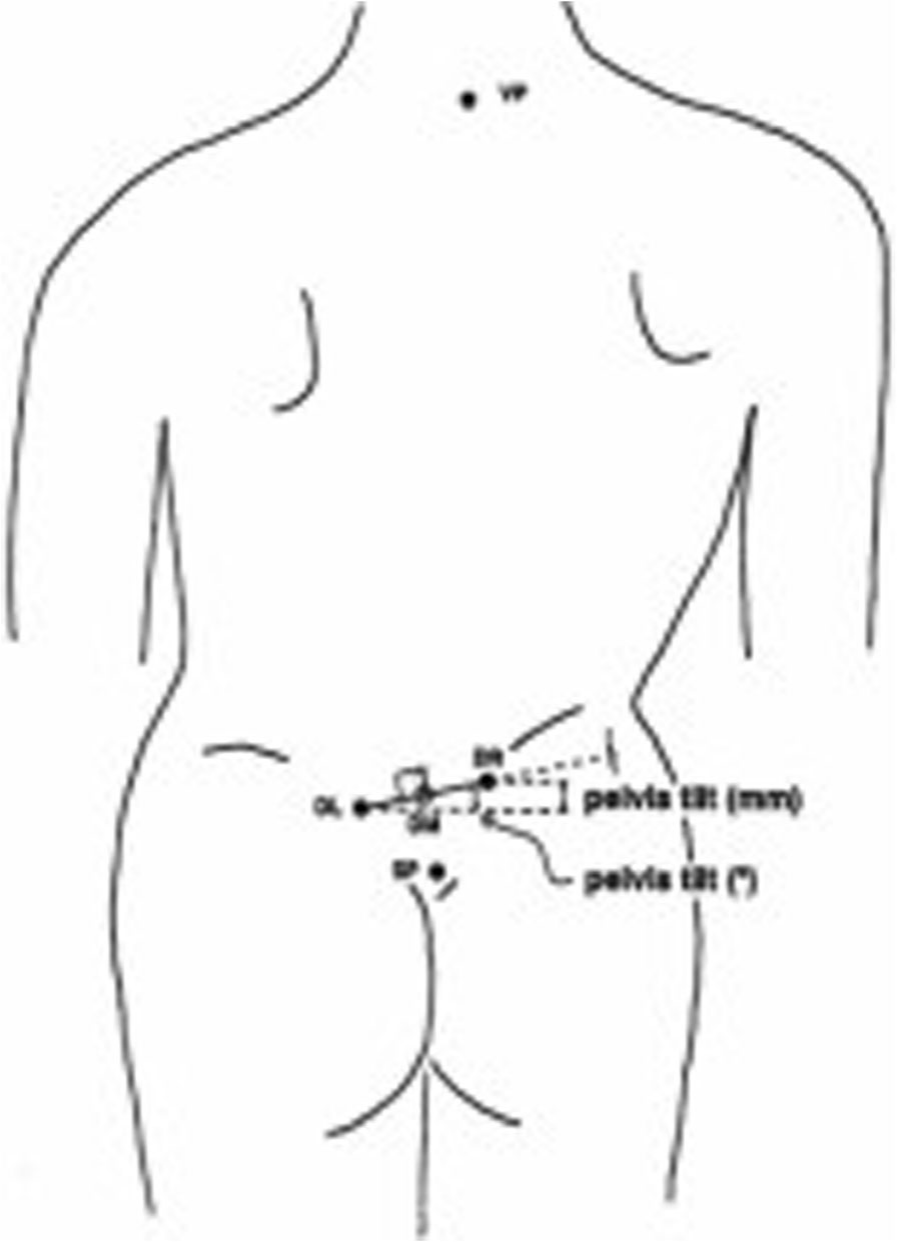
(From 4D Formetric Diers manual with permission). The pelvic tilt refers to a difference in height of the lumbar dimples, based on a horizontal plane

The following parameters were assessed (see also Table 1):

- *In coronal plane*: the coronal imbalance, Fig. 2, the pelvic obliquity/tilt, Fig. 3, the lateral deviation, and the 4DF scoliosis angle
- *In sagittal plane*: the sagittal imbalance/inclination, Fig. 2, the 4DF kyphotic angle, the kyphotic apex, the 4DF lordotic angle, the lordotic apex, the pelvic tilt, and the trunk inclination
- *In the transverse plane*: the pelvis rotation, the pelvic torsion, the surface rotation and the 4DF vertebral rotation to the right (deemed +), and 4DF vertebral rotation to the left (deemed −).

**Table 1:** Values measured using the proper software of the apparatus 4D Formetric DIERS (4DF) in the three cardinal planes

Coronal view	Min	Max	Mean value	SD
Coronal imbalance	0 mm	46 mm	11.4 mm	11.6
Pelvic tilt/obliquity	0 mm	15 mm	6.6 mm	4.4
Lateral deviation	0.7 mm	12.9 mm	6.4 mm	3.2
Scoliosis angle	5°	59°	17.5°	10.9
Sagittal view	Min	Max	Mean value	SD
Sagittal imbalance	− 1°	11	4.3°	3.4
Kyphotic angle	31°	61°	41.5°	7.6
Kyphotic apex	70 mm	236 mm	146 mm	52.0
Lordotic angle	15°	51°	35.3°	9.2
Lordotic apex	264 mm	412 mm	324.1 mm	38.7
Trunk inclination	0.1°	11.5°	4.5°	3,3
Transverse view	Min	Max	Mean value	
Pelvic rotation	0°	7°	2.5°	2.0
Pelvic torsion	0°	6°	2.3°	1.8
Surface rotation	1.1°	14.1°	5.0°	3.0
Vertebral rotation	3°	51°	12.5°	9.9

### Clinical measurements

The limbs’ length was clinically measured by a measuring tape with 0.1-cm increments, from the anterior superior iliac spine to the medial malleolus. The trunk asymmetry was recorded at mid-thoracic (T4–T8), thoracolumbar (T12–L1), and lumbar (L2–L5) region by performing the standing forward bending test using the Pruijs scoliometer which was supplied by Orthomet-Surgeyplant B.V. (Waalwijl, Netherlands). The child was asked to bend forward, looking down, keeping the feet 15 cm apart, knees braced back, shoulders loose, and hands positioned in front of knees or shins with elbows straight and palms opposed. The side of the hump determined the laterality of trunk rotation. Trunk asymmetry (hump) to the right side was defined as right asymmetry and to the left was defined as left asymmetry in each of the three mentioned regions and recorded in degrees.

In this series of children suffering LLI, the pelvic tilt value was corrected with the proper shoe elevation, corresponding to the right-left leg difference of the tape measurements. Subsequently, all the children were scanned again using the 4DF, aiming a pelvic tilt reading equaled to zero, that is, a horizontal pelvis and practically an equal legs’ length.

### The statistical analysis

For the Statistical Analysis (SA), the SPSS package v.22.0 was used. The techniques utilized were the descriptives, the deviation from the zero, ANOVA, and the LLI and scoliosis correlations among pelvic and spinal parameters.

### The reliability study of LLI measurements using the tape

In ten children, the right leg length was measured two times from the anterior iliac spine to the medial malleolus by TBG. KA measured the right leg length as well in the same children, using the same methodology. The difference for each pair (first and second measurement of TBG) of the right leg length and its SD was then calculated. Finally, the intra-observer error was calculated as 95% confidence limits using the formula:

$$ \mathrm{Intraobserver}\ \mathrm{error}:= \frac{\mathrm{SD}}{\sqrt{2}}x2 $$

The inter-observer error was calculated using the first set of TBG readings and those of KA, using the same formula.

## Results

The values of the studied parameters from the 4DF readings are presented in Table 1.

It was shown that the LLI was statistically significantly correlated to the 4DF readings of *pelvic obliquity*/*tilt* (*r* = −,674, *p* < .002) and the root mean square (rms) of 4DF reading surface rotation (*r* = −,533, *p* < .019). The scoliometer reading of angle of trunk rotation (ATR) in the lumbar level was statistically significantly correlated to the 4DF readings of pelvic tilt (*r* = −,629, *p* < .004) and rms surface rotation (*r*=.539, *p* < .017).

Doing ANOVA, it was shown that LLI was significantly associated to the sagittal imbalance in millimeters (*p* < 0.026) and to the trunk inclination in degrees (*p* < 0.026). Interestingly, it was shown that there was a lack of correlation of LLI to scoliosis angle.

The LLI reliability study showed an intra-observer of 0.08 cm and inter-observer error of 0.1 cm respectively.

## Discussion

A number of previous publications using radiographic methods reported on the effect of LLI on the spine and the posture [3, 9]. In literature, however, there is a paucity of reports, using surface topography, for the assessment of truncal and spinal changes due to mild LLI in growing children. So far, only simulating LLI studies in normal subjects have been published, assessing the effect of LLI on the spinal posture, the pelvic position, and on pelvic torsion and trunk mobility [11, 13].

This is the first report on the use of surface topography for the study of the effect of LLI on pelvic imbalance, spinal posture, and scoliotic curve in children suffering LLI.

The finding of this study, that is, a lack of correlation of LLI to the scoliosis angle, is in line with the findings presented in earlier reports studying this condition using radiological methods [1, 9, 10].

In LLI, the longer leg is usually responsible for the creation of the lumbar hump; therefore, the significant correlation of pelvic obliquity/tilt and of surface rotation to LLI makes sense. This is in line with the correlation of the scoliometer reading of the angle of trunk rotation (ATR) in the lumbar region to the 4DF readings of pelvic tilt and surface rotation. The convexity of lumbar scoliosis and the hump is at the side of the shorter leg. Infrequently, however, the convexity of lumbar scoliosis and the lumbar hump is present at the longer leg side, and this is also reported and explained why it happens, in the literature, [10]. Doing ANOVA, it was shown that LLI was significantly associated with the sagittal imbalance in millimeters (*p* < 0.026) and to the trunk inclination in degrees (*p* < 0.026).

Interestingly, it was shown that there is a lack of correlation of LLI to the scoliosis angle. This finding may indicate that scoliosis in LLI is an initial compensatory condition in growth and not a structural deformity. This also implies that for the development of structural scoliosis, apart from the existing LLI, more other aetiological factors might be present.

As noted in the “Introduction” section, the aim of this study is to present the effect of LLI on the presented parameters at first examination of a series of children suffering LLI and to provide information for the pelvic imbalance, spinal posture, and scoliotic curve, using Surface Topography Analysis.

The surface topography system (4D Formetric), takes the symmetry as normality. Especially in the coronal plane for the coronal balance, the pelvic obliquity, and the lateral deviation, the readings are expected to be zero in normality. Similarly, in the sagittal plane for the sagittal balance, the pelvic tilt and the trunk inclination, and in the transverse plane, for the pelvic rotation, the pelvic torsion, the surface rotation, and the 4DF vertebral rotation, the readings are also expected to be zero in normality.

The authors’ initial clinical interest was to make the pelvis horizontal by the application of shoe elevation in the shorter leg equal to right–left leg length difference. For this reason, in this study, information is not provided about the changes (corrections) of all the affected set of studied parameters in coronal, sagittal, and transverse view after the application of shoe elevation. The absence of information on the changes of the affected set of the studied parameter in coronal, sagittal, and transverse view is probably one of the limitations of this study, but the authors intend to present the long-term results of this patients’ group when these children and adolescents will be near adulthood in a few years. For a few of them, this period of time is not far away. It makes sense to us to look at the long-term outcomes, seeing that in terms of functional outcomes, such as posture or gait, persons who have developed a LLI later in life are more debilitated by LLI of the same magnitude when compared to persons who have had LLI since childhood [15].

The novel conclusion in this study, compared to previous studies, is that we recommend the correction of the mild LLI below the classic definition of 2.0 cm with the use of shoe elevations, as the parameters measured were statistically significantly changed and influenced pelvic imbalance and spinal posture. We also recommend the continuous follow-up of these patients until maturation and examination with raster-stereography (surface topography), as this method has many advantages, namely reduces the need for radiation exposure, provides a three dimensional quantification of posture, can be used to compare changes during follow-up assessment, and creates a completely harmless imaging set.

This report may be a useful basis for further understanding of truncal changes due to limb length discrepancy in adulthood as well.

## Abbreviations

FLLI: Functional limb length inequality
LLD: Limb length discrepancy
LLI: Limb length inequality
SLLI: Structural limb length inequality
